# Exhaled Nitric Oxide Fraction as an Add-On to ACQ-7 for Not Well Controlled Asthma Detection

**DOI:** 10.1371/journal.pone.0077085

**Published:** 2013-10-25

**Authors:** Vicente Plaza, David Ramos-Barbón, Ana María Muñoz, Ana María Fortuna, Astrid Crespo, Cristina Murio, Rosa Palomino, on behalf of the EOLO Study Investigators

**Affiliations:** 1 Servicio de Neumología, Hospital de la Santa Creu i Sant Pau, Institut d’Investigació Biomédica Sant Pau (IIB Sant Pau), Universitat Autònoma de Barcelona, Barcelona Respiratory Network (BRN), Barcelona, Spain; 2 Medical Department, Chiesi Spain, L’Hospitalet de Llobregat, Barcelona, Spain; 3 Área de investigación aplicada, GOC Networking, Barcelona, Spain; The Hospital for Sick Children and The University of Toronto, Canada

## Abstract

**Background:**

The measurement of fractional nitric oxide concentration in exhaled breath (FeNO), a noninvasive indicator of airway inflammation, remains controversial as a tool to assess asthma control. Guidelines currently limit asthma control assessment to symptom and spirometry based appraisals such as the Asthma Control Questionnaire-7 (ACQ-7). We aimed at determining whether adding FeNO to ACQ-7 improves current asthma clinical control assessment, through enhanced detection of not well controlled asthma.

**Methods:**

Asthmatic subjects, classified as not well controlled as per ACQ-7 on regular clinical practice, were included in a prospective, multicenter fashion, and had their maintenance treatment adjusted on visit 1. On follow-up (visit 2) four weeks later, the subjects were reevaluated as controlled or not well controlled using ACQ-7 versus a combination of FeNO and ACQ-7.

**Results:**

Out of 381 subjects enrolled, 225 (59.1%) had not well controlled asthma on visit 2 as determined by ACQ-7, and 264 (69.3%) as per combined FeNO and ACQ-7. The combination of FeNO to ACQ-7 increased by 14.8% the detection of not well controlled asthma following maintenance therapy adjustment.

**Conclusions:**

The addition of FeNO to ACQ-7 increased the detectability of not well controlled asthma upon adjustment of maintenance therapy. Adding a measure of airway inflammation to usual symptom and spirometry based scores increases the efficacy of current asthma clinical control assessment.

## Introduction

Chronic, diffuse inflammation affecting all airway categories is a cardinal feature of asthma pathophysiology. Therefore, the interest in assessing airway inflammation has led during recent years to the development of noninvasive procedures to measure inflammation-related indicators, notably inflammatory cell counts in induced sputum and the fractional nitric oxide concentration in exhaled breath (FeNO). For the latter, wide acceptance has been achieved for regular clinical practice, particularly in pediatric pulmonology, due to its ease of performance, low cost, and readily available readout. FeNO measurements deliver acceptable sensitivity and specificity for the diagnosis of asthma in non-smoker subjects devoid of inhaled corticosteroid therapy [1], particularly if their FEV_1_ is decreased. The procedure to perform FeNO measurements has been standardized [2] and its value ranges have been recently revised for better interpretability in clinical practice.

However, a debate on the utility of FeNO in asthma management is still ongoing. Other than few exceptions [3], mainstream asthma management guidelines have not established any use of FeNO for the diagnosis of asthma, nor to assess asthma control level [4]. Currently available evidence on FeNO performance to determine asthma control is ambivalent. Some studies have suggested that treated asthma patients who have high FeNO values suffer from more severe disease [5], have poorer asthma control, and are at greater risk for asthma exacerbations [6-8]. Conversely, other studies showed that the addition of FeNO measurements to the determinations usually employed to evaluate asthma control [9,10] and adjust therapy [11], such as standardized symptom questionnaires and spirometry, does not result in a better assessment of the current asthma control level nor the risk of exacerbation. A systematic review, which included a Cochrane based meta-analysis on six controlled randomized studies (two on adults and four on children/adolescents), showed data discrepancy in adults versus children and concluded that current evidence is not supportive on the use of FeNO for treatment adjustment [12]. More recently, a new meta-analysis of three adult studies comparing asthma exacerbation rates with FeNO-based versus clinically-based asthma management algorithms, one of which was not included in the former Cochrane meta-analysis, demonstrate that the rate of exacerbations was significantly reduced in favor of FeNO-based asthma management [13].

The usefulness of FeNO to determine the current asthma clinical control levels remains therefore controversial, due to the lack of solid evidence to definitely recommend or rule out such practice. In the work presented here, we aimed at contributing further data to better clarify the role of FeNO in asthma management. Our goal was to determine whether, as part of regular clinical practice, the inclusion of FeNO as an add-on measurement to symptom evaluation by the Asthma Control Questionnaire-7 (ACQ-7, which includes FEV1), improves the efficacy of current clinical asthma control assessment in comparison with ACQ-7 alone.

## Methods

### Legal and ethical aspects

The study was conducted in accordance with the Declaration of Helsinki principles (18th Word Medical Assembly, 1964) and was approved by an institutional Clinical Research Ethics Committee. The ethics approval number was CHI-EPI-2008. The participants provide their written informed consent to participate in this study. Subjects were included upon informed consent and personal identification data were anonymized.

### Study design

This was a prospective, multicentered, clinical-epidemiological study executed in 15 participating centers (13 Respirology and 2 Allergology departments) across Spain. Data were collected during regular clinical practice and medical procedures. The study was designed to compare the efficacy of FeNO in addition to ACQ-7, versus ACQ-7 alone, to determine the level of asthma control upon treatment adjustment in patients with previously not well controlled asthma.

### Study population

Male and female subjects, aged >17 years, with not well controlled persistent asthma and a positive bronchodilator test, a daily peak expiratory flow (PEF) variability greater than 20%, or a positive methacholine challenge test documented in case history, were included in the study. Asthma severity was defined as per the Global Initiative for Asthma Management (GINA) [4] and Guía Española para el Manejo del Asma (GEMA) [3], and not well controlled asthma was defined as an ACQ-7 score equal to, or greater than, 0.75 on visit 1. Pulmonary function testing was performed and interpreted according to European Respiratory Society/American Thoracic Society guidelines. Patients were excluded from the study if they were active smokers; had a respiratory tract infection or asthma exacerbation within 30 days prior to inclusion; had concomitant disease with a potential to alter FeNO level (sarcoidosis, lung cancer, pulmonary tuberculosis, bronchiectasis, nephropathy, rheumatic or liver disease); significant comorbidity that could alter the study results upon investigator's judgment; programmed hospitalization during the study; or a cognitive disorder that could limit the subject's study comprehension or collaboration. 

### Assessments and outcomes

Participating subjects were evaluated through two sequential visits. On visit 1, the subjects were included in the study and their asthma treatment adjusted as needed upon current asthma control. Recruitment was done at hospital outpatient clinics or specialist consult at primary health centers. Visit 2 followed four weeks later. On both visits, spirometry, ACQ-7 and FeNO were performed. Spirometry was performed according to the European Respiratory Society/American Thoracic Society guidelines using the predicted values for Mediterranean populations established by Roca et al [14,15]. The ACQ-7, a patient self-assessment questionnaire designed to evaluate asthma control from symptoms and pulmonary function [16], was used in its validated Spanish version [17]. The questionnaire contains 7 items comprising 6 multiple-choice test questions on the frequency of asthma symptoms and the use of rescue medication within the prior 7 days, and the FEV_1_ percent of predicted value. The total ACQ-7 score, computed from its 7 items, ranges from 0 (maximum control) to 6 (minimum control), and a 0.75 point threshold was chosen to consider controlled asthma [16]. FeNO was measured before spirometry, using NioxMino® portable equipment (Aerocrine, Sweden) and an expiratory maneuver providing a sustained 50 mL/s flow from total lung capacity [18]. Asthma was considered as controlled from the standpoint of airway inflammation if the FeNO value was less than 30 parts per billion (ppb) [3].

The primary study outcome was the difference in the proportion of patients with controlled asthma as per combined ACQ-7 and FeNO, compared to ACQ-7 alone, on visit 2. Secondary variables collected on visit 1 were demographics, anthropometric data, asthma severity according to GEMA [3], and number of asthma exacerbations for the prior 6 months. FEV1 percent of predicted value, FeNO and ACQ-7 score were recorded for analysis as secondary variables on both visits.

### Sample size calculation and statistical analysis

Sample size was calculated for the comparison of two related proportions on the basis of prior data on the detection of controlled asthma using ACQ-7 and FeNO [19], and resulted in 333 subjects to achieve a 95% statistical power with a level of significance of 5% for bilateral contrast, assuming a 10% proportion of discordant pairs. A 20% loss to follow-up was predicted since the study was prospective, therefore leading to a total of 424 subjects to be included in visit 1. Subjects were included in the study on consecutive random sampling basis, and each participating center had a recruitment compromise of 30 patients minimum completing visit 2. 

Categorical variables are expressed as absolute and relative frequencies, and quantitative variables as mean and standard deviation. Comparisons between visit 1 and visit 2 data sets were analyzed using paired-data student's t-test or non-parametric analysis for quantitative or ordinal variables as appropriate, or chi-square or McNemar test for categorical variables. Sensitivity, specificity, positive predictive value and negative predictive value were calculated for the ACQ-7 plus FeNO combination versus ACQ-7. A Receiver Operating Characteristics (ROC) was generated, and the area under the curve estimated. Correlation was evaluated with Spearman's coefficient. A *P* value of less than 0.05 was considered for statistical significance. Analysis was performed with SPSS software version 18.0 (SPSS Inc., Chicago, IL, USA). 

## Results

Four hundred and ninety subjects were considered for the study, of which 109 were excluded due to ACQ-7 or FeNO missing data, or not fulfilling inclusion criteria. Three hundred and eighty one assessable subjects with complete data were included in the statistical analysis. Table 1 shows the demographics and asthma characteristics for the subjects analyzed. 

**Table 1:** Demographic and asthma characteristics on visits 1 and 2.

	**Visit 1**	**Visit 2**	***P***
**Age**, years	44.3 ± 14.86	-	-
**Women**, %	57%	-	-
**Average number of asthma exacerbations in the prior 6 months**	1.62 ± 1.53	-	-
**Atopy**, **subjects** (%)*	204 (66.4%)	-	-
**Positive FEV_1_ bronchodilator response**, subjects (%)*	327 (85.8%)	-	-
**Daily PEF variability > 20%**, subjects (%)*	127 (33.3%)	-	-
**Positive methacholine challenge test**, subjects (%)*	51 (13.4%)	-	-
**Asthma Severity**		-	-
**- Intermittent asthma**, subjects (%)	21 (5.6%)		
**- mild persistent asthma**, subjects (%)	131 (35.2%)		
**- moderate persistent asthma**, subjects (%)	179 (48.1%)		
**- severe persistent asthma**, subjects (%)	41 (11%)		
**FEV_1_, % of predicted**	79 ± 18.8	85.3 ± 16.6	<0.001
**ACQ-7 score**	2.21 ± 0.81	1.10 ± 0.78	<0.001
**FeNO** (ppb)	43.18 ± 29.82	26.8 ± 20.82	<0.001

* Documented in clinical records. Values are mean ± standard deviation or number and percentage of subjects, as indicated.

Table 2 shows the FeNO and ACQ-7 (including FEV_1_) values collected to evaluate asthma control on visits 1 and 2, and the numbers and percentages of subjects categorized as having not well controlled or controlled asthma. Following maintenance therapy adjustment on visit 1, the FeNO and ACQ-7 values were significantly reduced on visit 2. The proportion of not well controlled patients was also significantly decreased on visit 2 for whichever classification criterion employed, whether ACQ-7, FeNO or both combined. On visit 2, the combination of ACQ-7 and FeNO detected 39 additional cases as not well controlled in comparison with ACQ-7 alone, which corresponds to a 14.8% increase in the detection of not well controlled patients. Table 3 shows patient distribution as controlled or not well controlled asthma on visit 2, classified as per FeNO plus ACQ-7 score versus ACQ-7 alone. The data were also analyzed on the patients classified as atopic versus non-atopic. This analysis showed that the addition of FeNO to ACQ-7 yielded a 10.8% increment of patients classed as not well controlled asthma on visit 2 in the atopic group and, similarly, 8.7% in the non-atopic group. 

**Table 2:** Evaluation of asthma control.

	**Visit 1**	**Visit 2**	***P***
**Not Well Controlled Asthma**			
**Not well controlled asthma as per FeNO, subjects (%)**	247 (64.8)	115 (30.2)	<0.001
**Not well controlled asthma as per ACQ-7, subjects (%)**	381 (100)	225 (59.1)	<0.001
**Not well controlled asthma as per FeNO +ACQ-7, subjects (%)**	381 (100)	264 (69.3)	<0.001
**ACQ-7 score in not well controlled subjects as per FeNO**	2.41 ± 0.85	1.23 ± 0.88	<0.001
**FeNO ppb in not well controlled subjects as per ACQ-7**	44.40 ± 31.49	28.60 ± 20.89	<0.001
**Controlled Asthma**			
**Controlled asthma as per FeNO, subjects (%)**	134 (35.2)	266 (69.8)	<0.001
**Controlled asthma as per ACQ-7, subjects (%)**	0 (0.0)	156 (40.9)	-
**Controlled asthma as per FeNO+ACQ-7, subjects (%)**	0 (0.0)	117 (30.7)	-
**ACQ-7 score in controlled subjects as per FeNO**	2.10 ± 0.81	1.05 ± 0.74	<0.001
**FeNO ppb in controlled subjects as per ACQ-7**	0 (0.0)	24.19 ± 20.52	-

**Table 3:** Subject asthma control distribution on visit 2 as per FeNO plus ACQ-7 versus ACQ-7 alone.

		**ACQ-7**		**TOTAL**
		**Not well controlled**	**Controlled**	
**FeNO + ACQ-7**	**Not well controlled**	225	39	264 (69.3)
	**Controlled**	0	117	117 (30.7)
**TOTAL**		225 (59.0)	156 (40.9)	381

Values are absolute number of subjects and percentages in parentheses.

**Figure 1:**
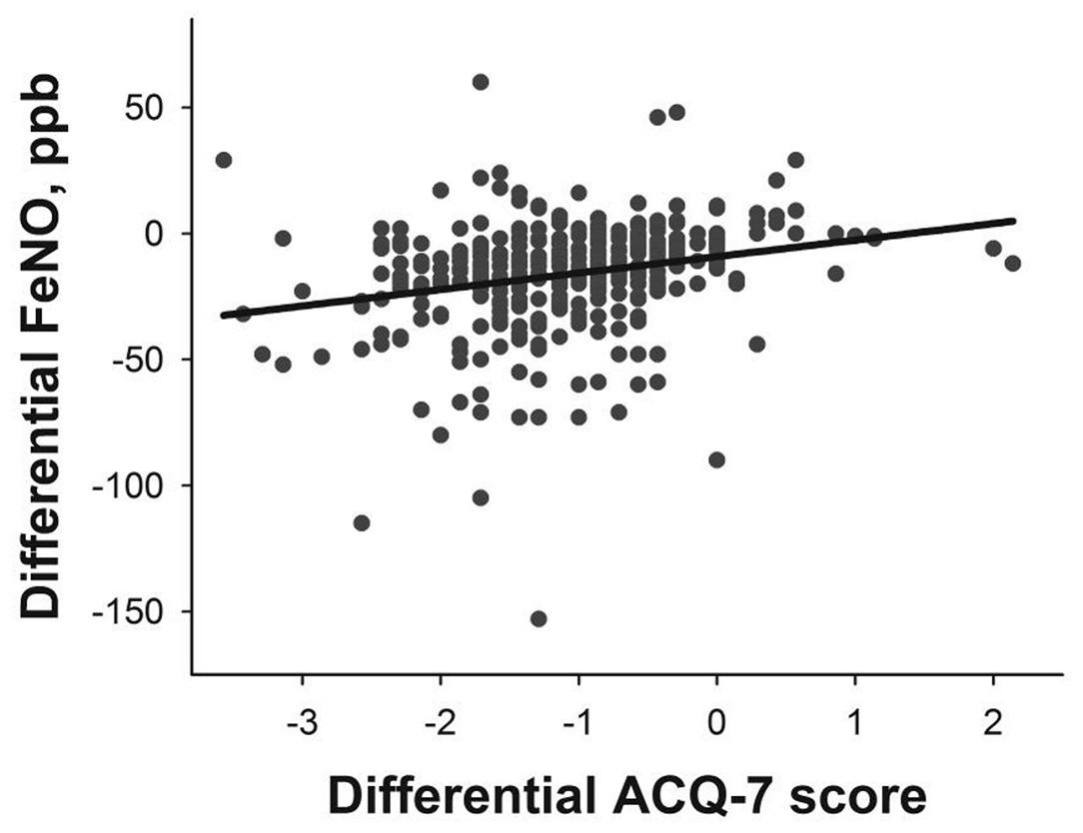
Correlation between the ACQ-7 score and FeNO change on visit 2 versus visit 1. The axes represent the ACQ-7 and FeNO differences on visit 2 minus visit 1, respectively. The magnitude of the decrease in the FeNO values showed a significant, positive correlation with the magnitude of the decrease in the ACQ-7 scores.

FeNO values and ACQ-7 scores correlated weakly on both visit 1 (r=0.150, *P*<0.01) and visit 2 (r=0.180, *P*<0.01). However, the decrease of FeNO and ACQ-7 from visit 1 to visit 2 showed a stronger correlation (r=0.309; *P*<0.001, Figure 1).

The combination of FeNO and ACQ-7, referenced to ACQ-7, showed 75% specificity and a positive predictive value of 85.2% to identify patients with not well controlled asthma on visit 2 (calculated from the values in Table 3). The area under the ROC curve was 0.8754 for FeNO and ACQ-7 combined, and 0.544 for sole FeNO (Table 4).

**Table 4:** Diagnostic properties of combined FeNO and ACQ-7 to identify not well controlled asthma, referenced to ACQ-7.

	**FeNO**	**FeNO + ACQ-7**
**Sensitivity**	33.8%	100%
**Specificity**	75 %	75%
**VPP**	66.1%	85.2%
**VPN**	44 %	100%
**Area under the ROC curve**	0.544	0.875

VPP, positive predictive value; VPN, negative predictive value.

## Discussion

Available evidence suggests that those asthmatics with greater eosinophilic inflammation, whether reflected as high eosinophil counts in peripheral blood or directly demonstrated in the airways, have worse asthma control and a greater risk of future exacerbations [20-22]. FeNO, as a noninvasive marker of airway eosinophilic inflammation, has shown consistency with the clinical outcomes predicted by eosinophil counts. In asthmatic children, FeNO values greater than 50 ppb following corticosteroid withdrawal are 71% sensitive and 93% specific to predict an increased risk of a subsequent loss of asthma control [23]. Other studies have recently shown, both in children and adults, that an increased FeNO is associated with a worse current asthma control and an increased risk of exacerbations, and is predictive of a better response to anti-inflammatory therapy [5,8,24,25]. However, FeNO determinations have shown inferior performance to evaluate asthma control in comparison with standardized symptom questionnaires, with or without spirometry values included [9,10]. Consistently with the prior reports, our data series showed that 30.2% of asthmatics were identified as not well controlled by FeNO on visit 2, as opposed to 59.9% identified by ACQ-7. Therefore FeNO, as a convenient noninvasive indicator of airway inflammation, may have its place in the rating of current clinical control of asthma as a measurement supplemental to the information provided by other data sources such as symptom questionnaires and pulmonary function tests. Coherently with this idea, the main outcome of the present study suggests that the combination of FeNO with ACQ-7 issues greater efficacy to determine current control in asthmatics under maintenance treatment. Indeed, FeNO combined with ACQ-7 allowed us to identify approximately 15% more cases of not well controlled asthma. The presence of such patients, seeming to have controlled asthma symptoms and an acceptable FEV_1_ but poorly controlled airway inflammation, may result from two opposing events. As argued in a prior report, the higher FeNO values in such patients may not reflect greater airway inflammation, but be a confounding factor resulting from sustained exposure to an allergen [11]. Conversely, the increased FeNO may be an actual indicator of more intense airway inflammation, not sufficiently controlled by the current maintenance treatment. The latter is supported by reports demonstrating that the correlation between asthma symptoms, pulmonary function tests and airway inflammation is limited, and that such determinations may reflect different disease domains [26-28]. Furthermore, our data showing a significant correlation between the FeNO and ACQ-7 decrements from visit 1 to visit 2, is consistent with the idea that the FeNO values actually reflect airway inflammation in this subpopulation of patients. Such correlation would be unlikely should the FeNO values result from factors other than airway inflammation.

Since the different procedures that can be employed to determine the level of asthma control are informative on partial aspects of the disease, some authors consider that an appropriate appraisal of asthma control should widely comprise different procedures, including indicators of airway inflammation, beyond standardized symptom questionnaires and spirometry. This idea is stressed by the fact that inflammation is a cardinal feature of the pathophysiology of asthma. Boulet et al. [29] developed an asthma control assessment tool that combined symptom scores and pulmonary function testing with a measurement of airway inflammation, the latter being eosinophil counts in induced sputum. More recently, Schatz et al. [30] tested a similar approach, simplified in practice by replacing induced sputum by a FeNO measurement. They also employed spirometry, a validated and widely diffused symptom questionnaire such as the Asthma Control Test (ACT), and the Asthma Intensity Manifestations Score (AIMS) from the Expert Panel Report-3 (EPR3) [31], which collects information on the needs of treatment. The AIMS was validated through a one-year follow-up on a sample of 304 asthmatics treated with inhaled corticosteroids. It scores from 0 to 4, and a ≥2 score is associated with worse asthma control, a two-fold risk of exacerbation and the four-fold risk of requiring courses of oral steroids during the following year.

Previous studies evaluating the utility of incorporating the FeNO to usual methods to determine the level of asthma control showed inconsistent results, from favorable [6,7,13,29,30] to negative [9-12]. Differences in the study design among the various reports may explain the variability of the results. Factors accounting for not detecting an added value for FeNO in the determination of asthma control, in some of the studies, may have been: (i) selecting a study population with a low probability of losing asthma control or undergoing exacerbations; (ii) running the study as a clinical trial, which may favor therapeutic adherence and therefore blunt the sensitivity of FeNO to cases with poor treatment compliance; and (iii) using the same tools, i.e. symptom questionnaires, to assess asthma control and guide therapy, and then as efficacy variables [32]. In regard to these limitations, our study presented here has two particular strengths: (i) the study was conducted under realistic, regular clinical practice conditions; and (ii) the selected study population had not well controlled asthma on study inclusion. A factor limiting the study was that, since the study was designed to evaluate current asthma control only, it lacked a further follow-up period to estimate the subsequent risk of asthma exacerbation.

In summary, our study presented here shows that, for a small but significant proportion of asthmatic subjects under maintenance therapy, assessing airway inflammation by means of FeNO supplements the information provided by the procedures currently recommended in the clinical guidelines, which are limited to symptom questionnaires and pulmonary function testing. Our data support the use of multidimensional tools to assess the level of current clinical asthma control. Such procedures should be informative on the different aspects of the disease, including symptoms, pulmonary function and airway inflammation.
